# A Nano-Micro Engineering Nanofiber for Electromagnetic Absorber, Green Shielding and Sensor

**DOI:** 10.1007/s40820-020-00552-9

**Published:** 2020-11-20

**Authors:** Min Zhang, Chen Han, Wen-Qiang Cao, Mao-Sheng Cao, Hui-Jing Yang, Jie Yuan

**Affiliations:** 1 School of Materials Science and Engineering, Beijing Institute of Technology, Beijing, 100081 People’s Republic of China; 2 Department of Physics, Tangshan Normal University, Tangshan, 063000 People’s Republic of China; 3 School of Information Engineering, Minzu University of China, Beijing, 100081 People’s Republic of China

**Keywords:** Electromagnetic absorber, Electromagnetic shielding, NiCo_2_O_4_ nanofiber, Sensor

## Abstract

**Highlights:**

- The role of electron transport characteristics in electromagnetic (EM) attenuation can be generalized to other EM functional materials.
- The integrated functions of efficient EM absorption and green shielding open the view of EM multifunctional materials.
- A novel sensing mechanism based on intrinsic EM attenuation performance and EM resonance coupling effect is revealed.

**Abstract:**

It is extremely unattainable for a material to simultaneously obtain efficient electromagnetic (EM) absorption and green shielding performance, which has not been reported due to the competition between conduction loss and reflection. Herein, by tailoring the internal structure through nano-micro engineering, a NiCo_2_O_4_ nanofiber with integrated EM absorbing and green shielding as well as strain sensing functions is obtained. With the improvement of charge transport capability of the nanofiber, the performance can be converted from EM absorption to shielding, or even coexist. Particularly, as the conductivity rising, the reflection loss declines from − 52.72 to − 10.5 dB, while the EM interference shielding effectiveness increases to 13.4 dB, suggesting the coexistence of the two EM functions. Furthermore, based on the high EM absorption, a strain sensor is designed through the resonance coupling of the patterned NiCo_2_O_4_ structure. These strategies for tuning EM performance and constructing devices can be extended to other EM functional materials to promote the development of electromagnetic driven devices.

**Graphic Abstract:**

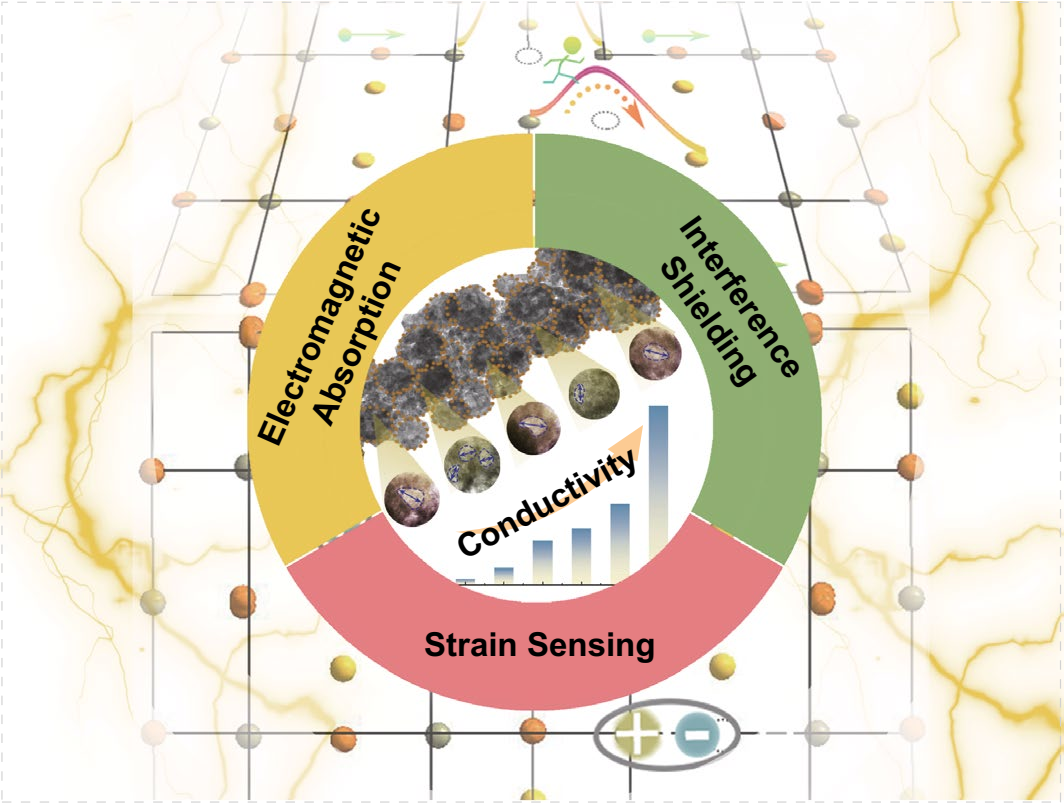

**Electronic supplementary material:**

The online version of this article (10.1007/s40820-020-00552-9) contains supplementary material, which is available to authorized users.

## Introduction

Porous nanostructures, with great potential for supercapacitors, electromagnetic (EM) attenuation, catalysis, and biological medicine, are attracting growing interest [1–11]. A thorough comprehension of the growth mechanism significantly facilitates the tailoring of porous morphology and analysis of material properties. Especially for EM properties, the superiority of porous nanostructures including lightweight, high specific surface, and rich electron transmission channels exposes the significance in promoting the innovation of EM functional materials.

EM functional materials have always been a hot spot in the information explosion era, and their applications in ultra-long-distance energy transmission, military stealth camouflage, and anti-interference of electrical equipment cannot be ignored [12–19]. Generally, the EM absorption mechanism fatefully depends on the impedance matching level, and incident EM wave is attenuated by dielectric or/and magnetic losses [20–28]. Many meritorious electromagnetic absorbing materials are emerging in recent decades, such as carbon materials, metal-based material, and polymers. [29–32]. Chen’s group conducted a series of studies to explore microwave absorbing materials [33–38]. Particularly, polyaniline and polypyrrole exhibit strong microwave absorption, while polyoxometalates and organic metal halide feature multi-band microwave absorption.

Compared with EM absorbing materials, the performance of shielding materials is contributed not only by effective absorption but by reflection [39–43]. The robust reflection caused by remarkable electrical property enables them to be integrated into contact lenses and clothing to protect life from EM radiation [44, 45]. However, the strong secondary reflection adds extra insecurity to the environment. Thus, “green” shielding materials emerge by tailoring the nanostructure to raise effective absorption but reflection [46]. It is unattainable for the EM absorption material to have the EM interference (EMI) shielding performance concurrently due to the feature that reflection and absorption are opposites, and conductivity (*σ*) simultaneously dominates conduction loss (*ε*_c_′′) and reflection. That is, there is a competition effect between conduction loss and reflection, and the balance between *ε*_c_′′ and reflection is hard to obtain.

Integrating EM property with electronic devices is an inevitable trend for innovation and breakthroughs in advanced electromagnetic devices [47]. However, such research associated with the EM multifunctional material or device is very limited, although EM functional materials are in a booming stage. The single function mode hardly captures the rapid development of EM devices.

Herein, a new dual-template nano-micro engineering is presented to tailor the internal structure of porous NiCo_2_O_4_ nanofiber. With customizing the template state, the polarization and charge transport properties are modulated. By analyzing the balance of *ε*_c_′′ and reflected EM wave, the shielding function is turned on by the “switch” of *σ*. Thus, EM absorbing and shielding can be achieved simultaneously. The mechanism of the state transition from EM absorption to shielding is gotten insight into. A patterned strain induction device, integrating the intrinsic EM absorption performance and resonance coupling effect of patterned structure, is designed. This sensor enables wireless and real-time detection of strain, potentially used in monitoring the state of large devices in harsh environments.

## Experimental Section

### Synthesis of NiCo_2_O_4_

The precursor was synthesized by electrospinning method. In particular, polyacrylonitrile (500 mg) and DMF (10 mL) were stirred for 12 h at 55 °C. 560 mg of Co(Ac)_2_·4H_2_O and 240 mg of Ni(Ac)_2_·4H_2_O were added to the mixture with stirring for 6 h. Then, the solution was injected into the syringe to start electrospinning. The feeding speed was set to 0.1 mm min^−1^, and the voltage was set to 16 kV. The as-spun precursor was achieved after dried for 12 h at 60 °C. The Ni-Co@C was synthesized by a two-step calcination method. Firstly, the precursor was heat treated at 180 °C for 2 h in N_2_. Then, it was heat treated at 500, 600, and 700 °C, respectively, for 2 h in N_2_. The Ni-Co@C nanofiber was obtained. Finally, porous NiCo_2_O_4_ nanofiber was heat treated at 400 °C in air.

### Fabrication of NiCo_2_O_4_ Composites

NiCo_2_O_4_ powders with different loading content (50, 70, and 90 wt%) were mixed with paraffin wax. Modest doses of C_4_H_10_O were added to the NiCo_2_O_4_-paraffin mixture with ultrasound until a uniform powder was obtained. Then, it was pressed into a toroidal EM mold. The inner diameter of the toroidal composite is 3.00 mm, and the outer diameter is 7.00 mm.

### Characterization

The morphology and microstructure of Ni-Co@C and NiCo_2_O_4_ were observed by scanning electron microscopy (SEM; HITACHI S-4800) and transmission electron microscopy (TEM; JEOL-2100). The crystal structure, elements, and composition were analyzed by an X-ray diffractometer (XRD; X’Pert PRO), X-ray photoelectron spectrometer (XPS; PHI Quanteral II (Japan)), Raman spectrometer (Renishaw Raman RE01), and Mettler Toledo thermal analysis TGA/DSC system.

### Calculation of Dielectric Properties

*ε*_p_′′ and *ε*_c_′′ are the dielectric loss contributed by polarization relaxation and charge transport, respectively, which can be obtained according to Debye theory (Eqs. 1–3):

1 $$\varepsilon^{\prime \prime } = \frac{{\varepsilon_{s} - \varepsilon_{\infty } }}{{1 + \left( {2\pi f} \right)^{2} \tau^{2} }}\omega \tau + \frac{\sigma }{{2\pi f\varepsilon_{0} }} = \varepsilon_{p}^{\prime \prime } + \varepsilon_{c}^{\prime \prime }$$

2 $$\varepsilon_{p}^{\prime \prime } = \frac{{\varepsilon_{s} - \varepsilon_{\infty } }}{{1 + \left( {2\pi f} \right)^{2} \tau^{2} }}\omega \tau = \varepsilon^{\prime \prime } - \varepsilon_{c}^{\prime \prime }$$

3 $$\varepsilon_{c}^{\prime \prime } = \frac{\sigma }{{2\pi f\varepsilon_{0} }}$$

where *ε*_s_ is the relative permittivity at static, and *ε*_∞_ is that at “infinite” high frequency. *τ* is the relaxation time. *f* is the frequency. *σ* is the DC conductivity, and *ε*_0_ is the vacuum permittivity.

### Calculation of Reflection Loss

The input impedance is calculated by Eq. 4:

4 $$Z_{\text{in}} = \sqrt {\frac{{\mu_{r} }}{{\varepsilon_{r} }}} \tanh \left[ {j\frac{2\pi }{c}\sqrt {\varepsilon_{r} \mu_{r} } fd} \right]$$

where *c* is the light velocity and *d* is the thickness of the sample. The reflection loss (RL) is calculated by Eq. 5:

5 $${\text{RL}}\left( {\text{dB}} \right) = 20\lg \frac{{\left| {Z_{\text{in}} - 1} \right|}}{{\left| {Z_{\text{in}} + 1} \right|}}$$

### Calculation of Electromagnetic Interference Shielding Performance

EMI shielding effectiveness (SE), SE_A_, and SE_R_ can be calculated by Eqs. 6–8,

6 $${\text{SE}}\left( {\text{dB}} \right) = 10 \times \lg \left( {1/T} \right)$$

7 $${\text{SE}}_{R} \left( {\text{dB}} \right) = 10 \times \lg \left( {1/\left( {1 - R} \right)} \right)$$

8 $${\text{SE}}_{A} \left( {\text{dB}} \right) = 10 \times \lg \left( {\left( {1 - R} \right)/T} \right)$$

### Calculation of *A*_eff_

The effective absorption efficiency *A*_eff_ is calculated by Eq. 9:

9 $$A_{\text{eff}} = \frac{1 - R - T}{1 - R}$$

where *R* and *T* are the reflection and transmission coefficient.

## Results and Discussion

### Structure Characterization

**Fig. 1:**
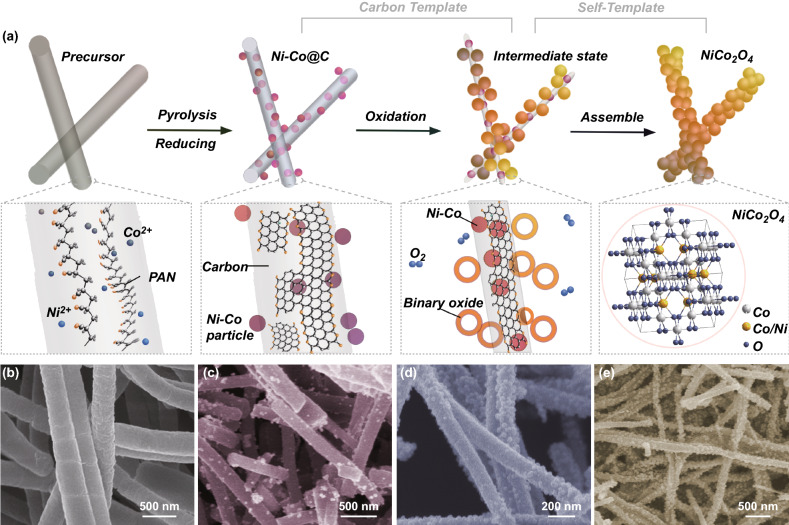
**a** Growth process for NiCo_2_O_4_, and the corresponding cross-sectional view with different molecular structure. **b-e** SEM images corresponding to each growth process

The growth process and compositional evolution of NiCo_2_O_4_ are shown in Fig. 1a. Precursor nanofibers composed of polyacrylonitrile (PAN) and metal ions (Ni^2+^:Co^2+^ = 1:2) are prepared by electrospinning strategy (Fig. 1b). After calcined for 4 h in N_2_, PAN nanofiber is carbonized with removal of non-carbon elements and Ni^2+^/Co^2+^ ions are reduced to Ni/Co, giving rise to the formation of Ni-Co@C nanofiber (Fig. 1c) [48, 49]. Then, the oxidation is proceeded by heating Ni-Co@C at 400 °C in air (Fig. 1a). C fiber is used as a template to support the oxidation and assembly of bimetallic particles. The SEM images in Fig. 1d, e show the in situ assembly process of oxide nanoparticles into a fibrous structure. Ni–Co particles on the nanofiber surface are oxidized first. Then, C fiber is gradually burned with the metal particles inside being exposed and oxidized continuously until the reaction is complete.

**Fig. 2:**
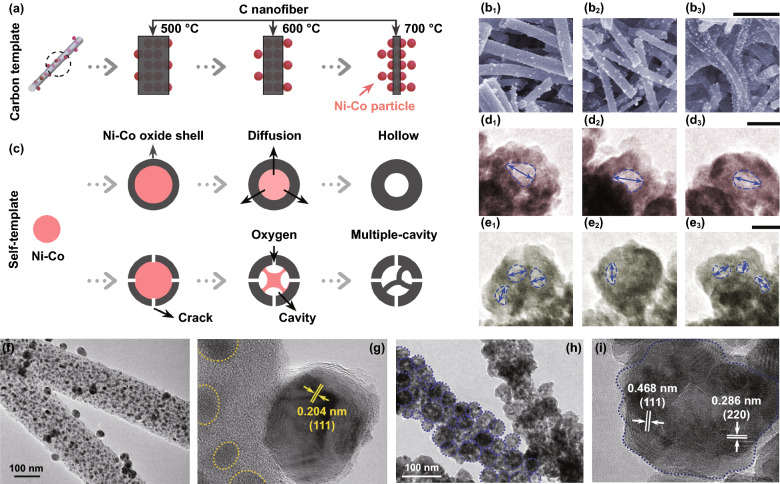
**a** Effect of the calcination temperature on the C template. **b C**orresponding SEM images. Scale bar is 1 um. **c** Schematic of different oxidation processes affected by the continuity of the oxide shell. **d** Morphologies of hollow particles with different diameter. Scale bar is 20 nm. **e** Morphologies of multiple-cavity particles. Scale bar is 20 nm. **f–g** TEM images of Ni-Co@C. **h–i** TEM images of NiCo_2_O_4_

When obtaining Ni–Co@C, calcination temperature can effectively tailor the C template size. In Fig. 2a, by raising the temperature, the purification of carbonized PAN continues, and the carbon consumption in reducing metal ions increases as the reaction progress more completely, resulting in thinner C nanofiber [48]. The corresponding morphology is revealed in Fig. 2b. The density of Ni-Co nanoparticles denoted by the bright white dots increases with the raised temperature, indicating that the thinner C fibers expose more metallic particles. The TEM images in Fig. 2f, g confirm that Ni–Co particles denoted by the dark spots are implanted on and inside the C fibers randomly, with the interplanar spacing of 0.204 nm, in accordance with the (111) planes of face-centered cubic. This suggests that Ni replaces part of cobalt atoms to form Fm-3 m NiCo_2_ alloy [50]. The final oxidation products corresponding to 500, 600, and 700 °C are denoted by N1, N2, and N3, respectively.

During the assembly of bimetallic oxide nanoparticles, there are two categories of the oxidation morphologies (Fig. 2c). When metal particles begin to oxidize, their edges preferentially nucleate to form a core–shell (metal–metal oxide) structure. The thin oxide shell acts as a template to support subsequent reactions. (i) For continuous shells, due to the Kirkendall effect that diffusion coefficient of Ni/Co is higher compared to oxygen, the central metal atoms can diffuse outward through the shell and are oxidized, generating a hollow particle with an expanding cavity [51–54]. Figure 2d shows the TEM images of hollow oxide particles with different diameters of 17.80, 18.61, and 16.69 nm. (ii) For shells with cracks, oxygen can enter the shell through the cracks, and the metal core is locally oxidized to form multiple cavities [54]. Figure 2e shows the presence of multiple-cavity in one oxide particle with the diameter ranging from 8.10 to 14.51 nm. Furthermore, because of oxygen adsorption-induced segregation effect, Co element is preferentially distributed on the outer edge of the shell rather than evenly distributed [55]. The TEM images in Fig. 2h show the overall morphology of the nanofibers assembled by the hollow/multilocular bimetallic oxide nanoparticles. Such porous nanofibers are characterized by high specific surface and abundant electron transmission paths, leading to the improvement of materials’ properties, especially the electrical property. Figure 2i shows that nanofibers are not smooth, and the interplanar spacings of the oxide nanoparticle are 0.468 and 0.286 nm, respectively, indexed to the (111) and (220) planes of NiCo_2_O_4_. The corresponding analyses of XRD, Raman, and XPS spectra are shown in Fig. S1 [56–60].

### Electromagnetic Absorption Performance

**Fig. 3:**
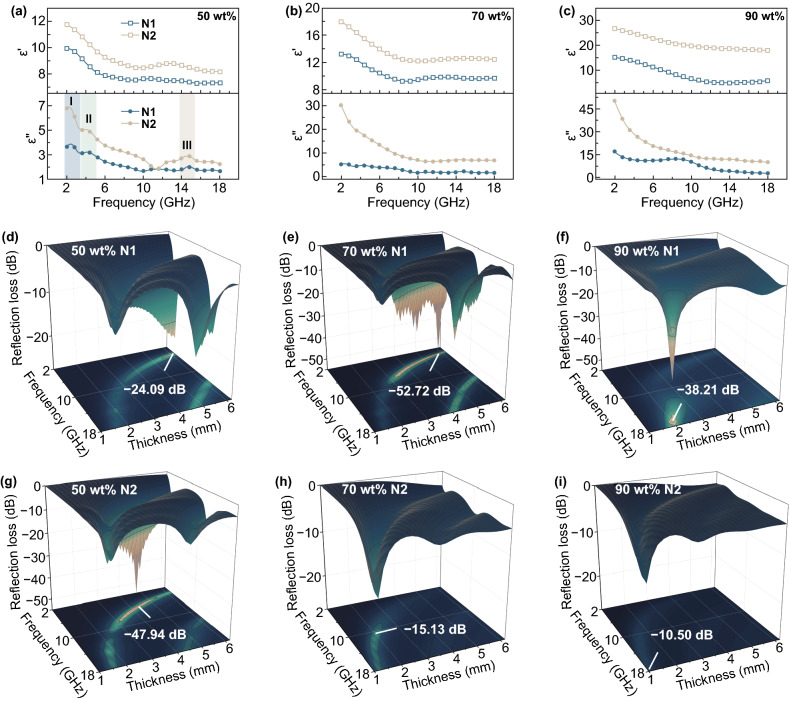
**a–c** *ε*′ and *ε*′′ of N1 and N2 composites with different loading concentrations of 50 wt%, 70 wt%, and 90 wt%. **d–f** RL of N1 with different loading concentrations. **g–i** RL of N2 with different loading concentrations

Since the fibrous morphology of N3 collapses (Fig. S2a–c), we only investigate the EM response of N1/N2. Figure 3a–c shows the EM responses of N1/N2-paraffin composites with loading content of 50, 70, and 90 wt%. As the loading content of NiCo_2_O_4_ rises, ε′ and ε″ increase. For each composite, *ε*′ is trending downward due to the declined conductance and polarization response at high frequency. For characterizing the dielectric loss *ε*′′, “material genes” of polarization and conduction loss (*ε*_p_′′ and *ε*_c_′′) are analyzed separately (Eqs. 1–3). *ε*_p_′′ is characterized by the relaxation peaks (I, II, and III). The multiple relaxation behaviors are arising from the inner friction of the dipole orientation polarization. *ε*_c_′′ is characterized by the declining trend of *ε*′′ due to the reduced conductance response. The relaxation peaks are becoming inconspicuous of 70/90 wt% composites owing to the relatively improved *σ* masking the peaks. Therefore, the synergy and competitive effect of polarization and conduction “genes” enable the modulation of the EM response. Figure 3d–i and S3 show that all composites possess dual absorption bands. The maximum RL reaches − 52.72 dB (70 wt% N1). The ordinary RL of 70/90 wt% N2 (− 15.13 and − 10.50 dB) originates from the growing reflection caused by the impedance mismatching between the material and free space.

**Fig. 4:**
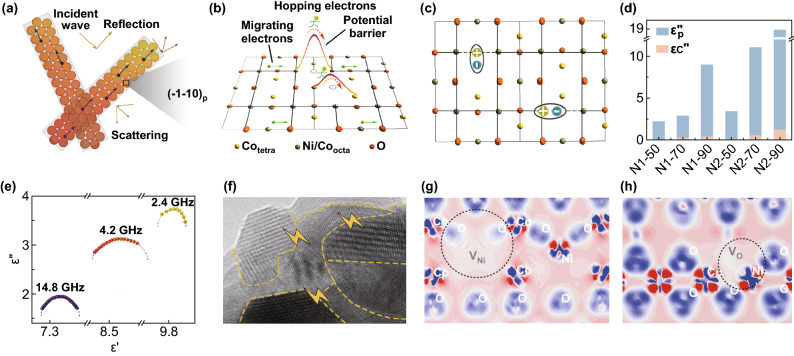
EM attenuation mechanism of NiCo_2_O_4_ nanofibers. **a** Multiple reflection and scattering. **b** Conductive network and charge transport. **c** Polarization induced by defects. **d** The contribution of *ε*_p_′′ and *ε*_c_′′ to *ε*′′. The suffixes − 50, − 70, and − 90 represent the loading concentration of the composites. **e** Cole–cole curves of 50 wt% N1. **f** Interfacial polarization observed from TEM image. **g, h** Difference charge density around Ni vacancy (*V*_Ni_) and oxygen vacancy (*V*_O_)

Figure 4 shows the EM attenuation mechanism of the composite. When dispersed in paraffin matrix, the one-dimensional NiCo_2_O_4_ and the rugged surface assembled by porous nanoparticles raise the probability of multiple reflections and scattering in the composite (Fig. 4a). In crystalline NiCo_2_O_4_, electrons absorb EM energy for transporting, including hopping the barrier and migrating in the network (Fig. 4b). The multi-cavity feature of NiCo_2_O_4_ nanoparticles not only reduces the density of the material, but also provides more channels for electron transmission, thus effectively improving the electron transmission efficiency. Figure 4d shows the ratio of the contribution of *ε*_p_′′ and *ε*_c_′′ to the dielectric loss. It reveals that NiCo_2_O_4_ is a polarization-dominated EM absorbing material, since the preparation process will introduce abundant defects and groups to form dipoles (Fig. 4c). The cole–cole curves of 50 wt% N1 in Fig. 4e are derived from the polarization relaxation peaks at ~ 2.4, 4.2, and 14.8 GHz. Figure 4f–h shows three different types of dipoles, including asymmetric charge distribution on phase boundaries with different atomic arrangements, Ni atom vacancy (*V*_Ni_), and oxygen vacancy (*V*_O_). In addition, there are other relaxation derived from metal ions of different valence states (Ni^2+/3+^, Co^2+/3+^) and impurity functional groups, which will attenuate EM energy by polarizing.

### Electromagnetic Interference Shielding Performance

**Fig. 5:**
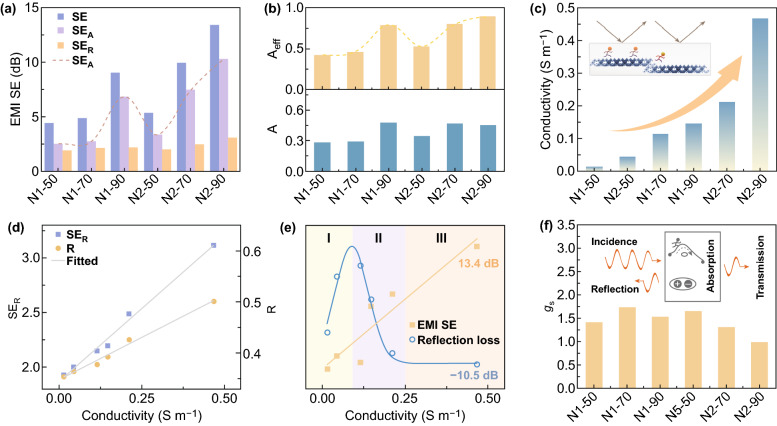
**a** Average EMI SE, SE_A_, and SE_R_ of different NiCo_2_O_4_ composites. **b** Average effective absorption and absorption coefficient of different composites. **c** *σ* of the NiCo_2_O_4_ composites. Sort in ascending order. Inset: schematic of the effect of σ on material properties. **d** SE_R_ and reflection coefficient versus *σ*. **e** Tendency of EM absorbing and shielding caused by charge transport. **f** *g*_s_ of each sample. Inset, schematic of EM wave incidence, reflection, transmission, and absorption

Generally, it is difficult for the EM absorption material to have the shielding performance, because the high *σ* of the shielding material will lead to enhanced reflection and weakened absorption. However, by tailoring the porous nanofiber structure, NiCo_2_O_4_ possesses an extremely high effective absorption, enabling the EMI shielding function. The average EMI SE in 2–18 GHz for different samples with a thickness of 3 mm is shown in Fig. 5a (Fig. S4). 90 wt% N2 has the maximum value of 13.44 dB. For all the samples, SE_A_ dominates the SE. Its change (dotted lines) is consistent with the effective absorption efficiency *A*_eff_ (Fig. 5b). *A*_eff_ of 70/90 wt% N1/N2 exceeds 50%, indicating that irrespective of reflection, EM wave is mostly used to support electron transport and establish polarization than transmission. When considering reflection, absorption coefficient (*A*) of 90 wt% N2 slightly decreases, because its high *σ* and strong reflection prevent some EM wave from entering the material. This is also why the samples of 90 wt% N1 and 70/90 wt% N2 with excellent *A* do not present high RL (Fig. 3). Samples are sorted based on *σ* in Fig. 5c. At the same loading content, *σ* of N2 is greater than N1. According to the aggregation-induced charge transport mode, the increase in *σ* with the loading content results from the improvement of the conductive networks and the stacking/contacting sites for electron hopping. Therefore, raising *σ* can improve *ε*_c_′′ so as to enhance *A*_eff_ as well as RL, and conversely, raising *σ* can strengthen reflection coefficient (*R*) and SE_R_ (Fig. 5d, and inset in Fig. 5c). Figure 5e shows the *σ*-driven EMI SE and RL has a competitive synergy. In region I, the increscent *σ* has a positive effect on EM absorption. In region II, RL gradually declines, whereas the EMI SE gradually increases. This is due to that *ε*_c_′′ and *R* will cause competition as *σ* contributes to the both. In region III, reflection dominates and promotes the EMI SE, where *σ* can be used as a switch to turn on the shielding performance. Here, EM absorbing and shielding of the NiCo_2_O_4_ composite exist simultaneously. In order to comprehensively evaluate the material’s eco-friendliness, the green shielding index (*g*_s_) is analyzed. According to $g_{s} = \frac{1}{{\left| {S_{11}^{2} } \right|}} - \frac{{\left| {S_{21}^{2} } \right|}}{{\left| {S_{11}^{2} } \right|}} - 1$ (where *S*_11_/*S*_21_ represents the reflection/transmission coefficient), a high *g*_s_ suggests a small secondary reflection and a large effective absorption. When the value is greater than 1, the secondary radiation pollution is less compared with the effective absorption. In Fig. 5f, for all samples $g_{s} \ge 1$, implying shielding performance mainly depends on the inherent attenuation rather than reflection. Furthermore, it should be noted that the shielding performance has the potential for further optimization, if novel strategies for precisely tailoring materials are developed to enhance *A*_eff_ and an optimal *σ* that balances *ε*_c_′′ and *R* is found. It is possible to achieve a prominent material with simultaneously high-efficiency EM absorbing and shielding, which is the challenge of the next work.

### Electromagnetic Sensor

**Fig. 6:**
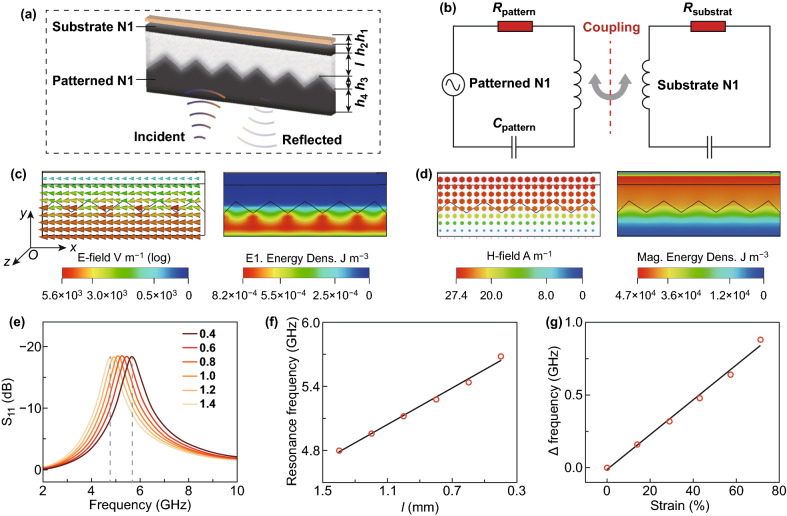
**a** Schematic illustration for the sensor. **b** Equivalent circuit model of the device. **c** Simulated electric field, electric energy density distributions. **d** Simulated magnetic field, magnetic energy density distributions. **e** Reflection spectra vs. frequency at *l *= 0.4–1.4 mm. **f** Distance-sensitive resonance frequency response. **g** Strain-response curves

The excellent EM performance allows us to conceive new functional materials and EM-driven devices. By integrating the resonant coupling effect of patterned structure into the intrinsic EM properties of NiCo_2_O_4_ composite, a strain sensor is designed to wireless record of pressure (Fig. 6a and S5). The structure can be equivalent to an electrical circuit, containing the resistance (R) of each component, the capacitance (C), and inductance (L) of zigzag pattern as well as the synergy of N1 and copper layers (Fig. 6b). The CST Microwave Studio for electric and magnetic field shows that the electric vectors resonate in the *x*-axis direction (Fig. 6c), while the magnetic vectors resonate in the *z*-axis direction (Fig. 6d). The corresponding energy density distributions demonstrate that electric component loss is concentrated in the patterned layer, and the magnetic component loss mainly occurs in the upper part due to the strong induced magnetic field by copper layer and N1 substrate. When the distance between the two N1 layers is compressed, the induced electric field caused by the magnetic vectors will overlap with the original electric vectors, thereby increasing the inductance coupling and modulating the response of reflection. Figure 6e shows the reflection spectra recorded from the structure. The resonance frequency shifts ~ 1 GHz by tuning the parameter *l* from 0.4 to 1.4 mm. Figure 6f shows that the resonance frequency is blue shifted and exhibits a linear response to *l* due to the raised inductance coupling. To construct a strain sensing EM device, the space between the two N1 layers is filled with a silicon rubber. When the silicon rubber is compressed with strain, the increment of the resonance frequency (∆Frequency) is proportional to the strain (*S*), ∆*f*_r_ ~ (0.012 ± 5E − 4)*S* (Fig. 6g). Compared to traditional sensors, this electromagnetic sensor with quick feedback shows greater competitiveness due to the ultrashort polarization establishment time of ~ 10^−9^ s. Thus, the strain sensor is a promising application in real-time and wireless pressure measurement for the 5G era.

## Conclusion

In summary, a multifunctional NiCo_2_O_4_ nanofiber is successfully fabricated via dual-template method. By controlling the template state, EM response of NiCo_2_O_4_ can be tuned. In particular, the increased charge transport capacity not only plays a dominant role in *ε*_c_′′ to promote EM attenuation, but reduces the degree of impedance matching, thus enhancing the EM reflection. Based on the results, the EM absorbing and green shielding functions of NiCo_2_O_4_ composite can be customized and coexistence. More importantly, a strain sensor device constructed by patterned NiCo_2_O_4_ composites is demonstrated. These findings open new horizons for design of multifunctional EM materials and will promote to expand the functions of NiCo_2_O_4_ to various fields, including metamaterials, sensing, and EM attenuation.

## Electronic supplementary material

Below is the link to the electronic supplementary material.

Supplementary material 1 (PDF 568 kb)
