# Supplementary material for: A Nano-Micro Engineering Nanofiber for Electromagnetic Absorber, Green Shielding and Sensor
Source: Nanomicro Lett. 2020 Nov 20;13:27. doi: 10.1007/s40820-020-00552-9 (PMC8187527; doi:10.1007/s40820-020-00552-9)
Supplement: Supplementary file 1 — Supplementary material 1 (PDF 568 kb) [file 40820_2020_552_MOESM1_ESM.pdf]

Supporting Information for

A Nano-Micro Engineering Nanofiber for Electromagnetic Absorber, Green Shielding and Sensor

Min Zhang[1, †], Chen Han[1, †], Wen-Qiang Cao[1], Mao-Sheng Cao[1, *], Hui-Jing Yang[2, *], Jie Yuan[3, *]

[1]School of Materials Science and Engineering, Beijing Institute of Technology, Beijing 100081, People's Republic of China

[2]Department of Physics, Tangshan Normal University, Tangshan 063000, People's Republic of China

[3]School of Information Engineering, Minzu University of China, Beijing 100081, People's Republic of China

†Min Zhang and Chen Han contributed equally to this work

*Corresponding authors.

E-mail: caomaosheng@bit.edu.cn (Mao-Sheng Cao); yanghuijing619@126.com (Hui-Jing Yang); yuanjie4000@sina.com (Jie Yuan)

Supplementary Figures

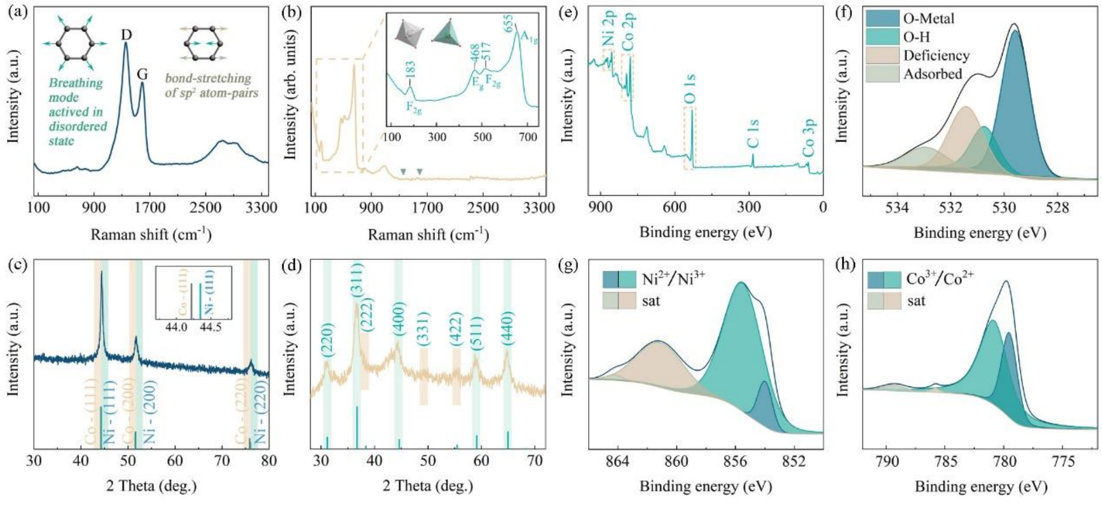

Fig. S1 Raman spectrum of (**a**) Ni-Co@C and (**b**) $NiCo_2O_4$. XRD patterns of (**c**) Ni-Co@C and (**d**) $NiCo_2O_4$. **e** XPS spectrum of $NiCo_2O_4$. **f** XPS of O 1s. **g** XPS of Ni $2p_{3/2}$. **h** XPS of Co $2p_{3/2}$

Figure S1a shows the Raman spectrum of C template. The D peak at 1353.5cm^{-1} corresponds to the breathing vibrations of six-fold aromatic rings, which is activated by disorder/defect. The G peak is located at 1590.7cm^{-1}, related to the stretching mode of sp^2 atom pairs in the plane [44, 45]. The intensity of D is significantly stronger than D, indicating a high structural disorder of C. Figure S1b shows the Raman spectrum of bimetallic oxide, where D and G peaks of C are

replaced by the characteristic modes of $NiCo_2O_4$, i.e., 183, 468, 517, and 655 cm^{-1}. The frequency at 655 cm^{-1} of A_{1g} mode involve breathing modes of the oxygen atoms in CoO_4 tetrahedra [46]. The E_g mode at 468 cm^{-1} originates from the bending motions of the oxygen atoms in tetrahedra. And the additional two F_{2g} modes at 183 and 517 cm^{-1} are associated with the order of Ni^{2+}/Co^{2+} in octahedron [47]. Figure S1c shows the XRD pattern of Ni-Co alloy. The diffraction peaks are consistent with the (111), (200), and (220) planes in Fm-3m space group of Ni/Co. And that in Fig. 3d demonstrates the cubic spinel phase of $NiCo_2O_4$. The XPS spectra of bimetallic oxide are shown in Fig. S1e-h. The O 1s spectra in figure S1f contain four oxygen species: (i) ions O^{2-} in the lattice and bonded to the metal (529.6 eV); (ii) ions O^{2-} in OH^- groups (530.7 eV); (iii) O species associated with the deficiencies (531.4 eV); and (iv) weakly adsorbed O species from air (533.0 eV) [48]. This result suggests that abundant defects and functional groups exist in the oxide sample. The $2p_{3/2}$ spectra of Ni and Co are fitted by different valence states and satellite (sat) signals, indicating Ni^{2+}, Ni^{3+}, Co^{2+}, and Co^{3+} coexist in the oxide sample (Fig. S1g-h). The ample defects and the diversity of ion valence states significantly increase the number of dipoles inside the material so as to enhance the number of dipoles and the dielectric property.

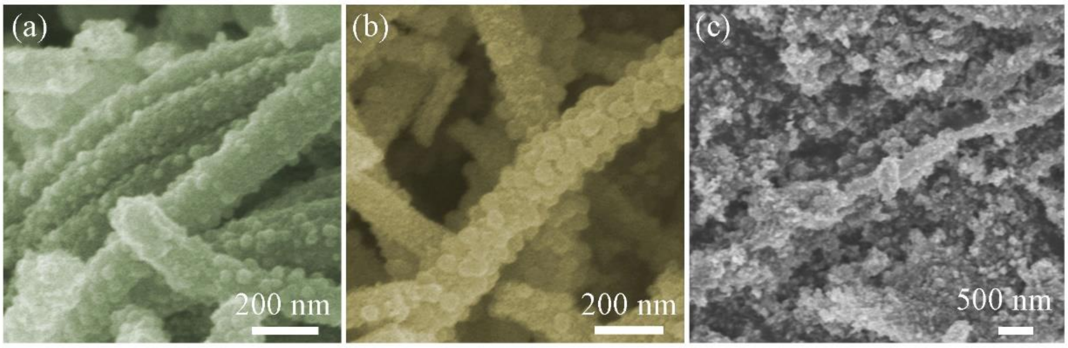

Fig. S2 a-c SEM images of N1, N2 and N3. The fibrous morphology of N3 collapses due to the too thin C template

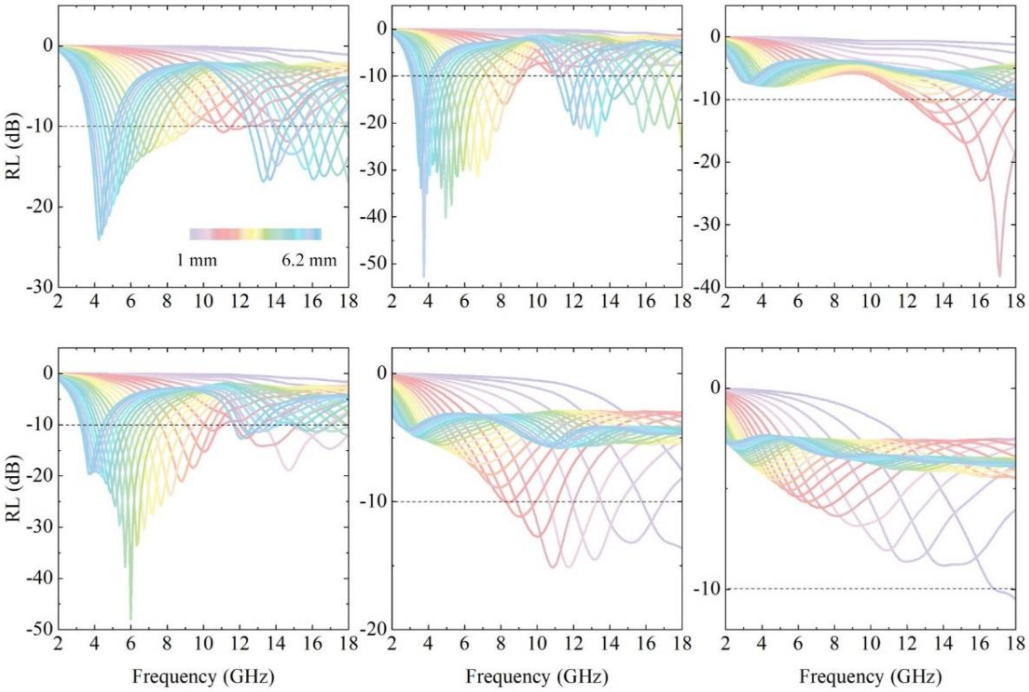

Fig. S3 Corresponding microwave absorption performance in Fig. 3d-i

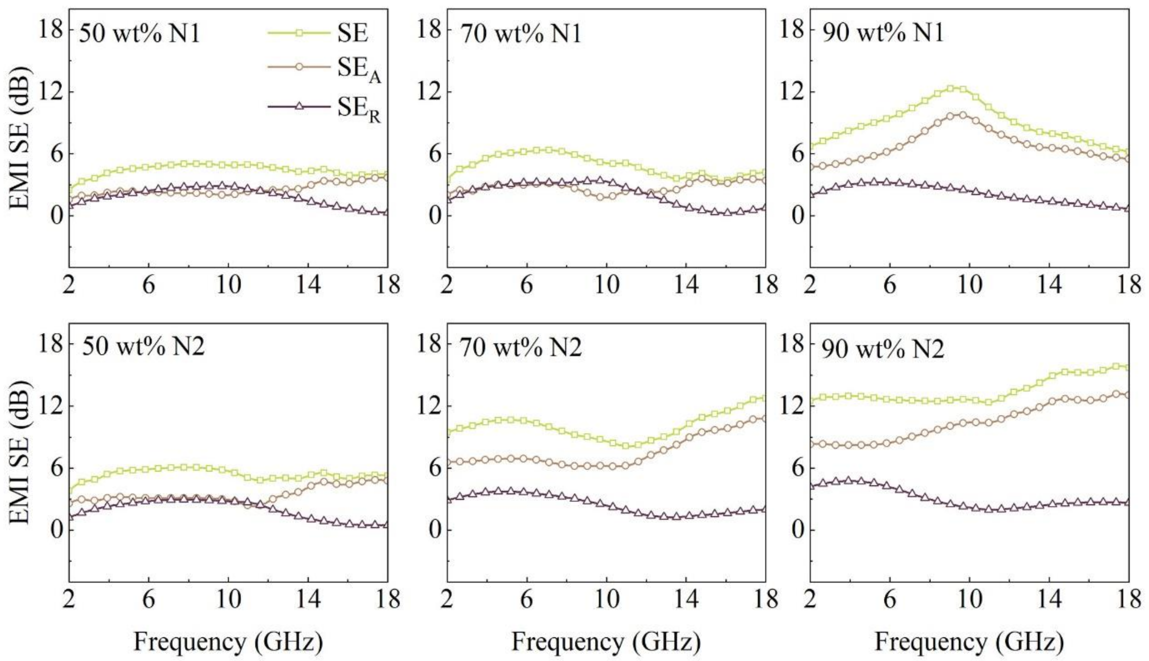

Fig. S4 EMI SE of the six $NiCo_2O_4$ composites

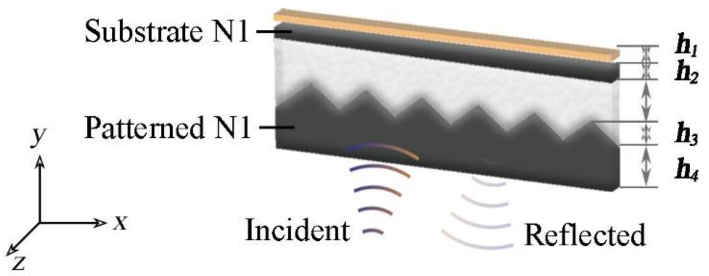

Fig. S5 Schematic illustration for $NiCo_2O_4$ composite patterns

The electromagnetic response of the patterned sensor is investigated by CST Microwave Studio. As shown in Fig. S5, a zigzag patterned 70wt% N1 composite (h_3=1 mm, h_4=2 mm) serves as the incident surface, and a 700 um (h_2) thick substrate of the same material is placed at a distance l. Copper (h_1=100 um) is selected as outer layer due to the mechanical and electrical superiority, such as high σ and strength. The incident wave port is towards the structure. The electric field boundary is set along x direction and the magnetic field boundary is set along z direction. The electric field, magnetic field, electric energy density and magnetic energy density monitors are exerted to reveal the field destructions inside the sensors. The sensor's working principle is that when the microwave incident on the sensor, the dielectric loss and coupled effect appear to attenuate electromagnetic wave. Meanwhile, the electromagnetic response is sensitive to the distance between two components, which can be to modulate the reflection coefficient S_{11}.
